# Biomonitoring of 2,4-Dichlorophenoxyacetic Acid Exposure and Dose in Farm Families

**DOI:** 10.1289/ehp.8869

**Published:** 2006-12-14

**Authors:** Bruce H. Alexander, Jack S. Mandel, Beth A. Baker, Carol J. Burns, Michael J. Bartels, John F. Acquavella, Christophe Gustin

**Affiliations:** 1 University of Minnesota, School of Public Health, Minneapolis, Minnesota, USA; 2 Emory University, Rollins School of Public Health, Atlanta, Georgia, USA; 3 University of Minnesota, School of Medicine, Minneapolis, Minnesota, USA; 4 The Dow Chemical Company, Midland, Michigan, USA; 5 Monsanto Company, St. Louis, Missouri, USA; 6 Amgen, Thousand Oaks, California, USA

**Keywords:** 2,4-D, biomonitoring, epidemiologic studies, herbicide, pesticide exposure

## Abstract

**Objective:**

We estimated 2,4-dichlorophenoxyacetic acid (2,4-D) exposure and systemic dose in farm family members following an application of 2,4-D on their farm.

**Methods:**

Farm families were recruited from licensed applicators in Minnesota and South Carolina. Eligible family members collected all urine during five 24-hr intervals, 1 day before through 3 days after an application of 2,4-D. Exposure profiles were characterized with 24-hr urine 2,4-D concentrations, which then were related to potential predictors of exposure. Systemic dose was estimated using the urine collections from the application day through the third day after application.

**Results:**

Median urine 2,4-D concentrations at baseline and day after application were 2.1 and 73.1 μ g/L for applicators, below the limit of detection, and 1.2 μ g/L for spouses, and 1.5 and 2.9 μ g/L for children. The younger children (4–11 years of age) had higher median post-application concentrations than the older children (≥ 12 years of age) (6.5 vs. 1.9 μ g/L). The geometric mean systemic doses (micrograms per kilogram body weight) were 2.46 (applicators), 0.8 (spouses), 0.22 (all children), 0.32 (children 4–11 years of age), and 0.12 (children ≥ 12 years of age). Exposure to the spouses and children was primarily determined by direct contact with the application process and the number of acres treated. Multivariate models identified glove use, repairing equipment, and number of acres treated as predictors of exposure in the applicators.

**Conclusions:**

We observed considerable heterogeneity of 2,4-D exposure among farm family members, primarily attributable to level of contact with the application process. Awareness of this variability and the actual magnitude of exposures are important for developing exposure and risk characterizations in 2,4-D–exposed agricultural populations.

2,4-dichlorophenoxyacetic acid (2,4-D) is a widely used herbicide with agricultural and nonagricultural applications. In the United States in 2001, 2,4-D ranked fifth among all pesticides in pounds of active ingredient applied in the agricultural market sector (28–33 million lb), the number one pesticide applied in both the home and garden market sector (8–11 million lb), as well as the industry, commercial and government market sector (16–18 million lb) (Donaldson et al. 2004). Studies of health outcomes related to chronic exposure to 2,4-D exposure have shown inconsistent results (Bukowska 2003; Burns et al. 2001; Figgs et al. 2000; Garabrant and Philbert 2002; Garry et al. 2001; Zahm et al. 1990), which may be attributed largely to difficulties with exposure characterization.

Unlike studies of industrial pesticide manufacturing workers, which often use personnel and industrial hygiene records to reconstruct exposure histories, epidemiologic studies of herbicide exposure in farmers, farmworkers, and farm families have generally relied on self-reported activity-based questionnaires to estimate exposure potential. The validity of questionnaire data for determining exposures within the agricultural populations has not been established, but a few studies that have addressed the issue indicate the validity is variable (Blair et al. 2002; Engel et al. 2001). Farmers report general pesticide usage practices with reasonable reliability, but the validity of reporting specific chemicals is problematic (Blair and Zahm 1993; Engel et al. 2001). The magnitude of exposure has especially been difficult to quantify for farm family members, including the primary applicator. Pesticide use is clearly the primary determinant of exposure for applicators, but characterizing exposure intensity within populations of applicators is difficult. Notable efforts to improve exposure estimates are often based on other exposure surrogates (Dosemeci et al. 2002). However, developing valid exposure models based on these surrogates is an elusive undertaking; such models must be developed with relevant biological monitoring data (Acquavella et al. 2006; Arbuckle et al. 2002). To better characterize exposure from herbicide use, we present biomonitoring results from the Farm Family Exposure Study (FFES) evaluating 2,4-D exposure in farm family members following a single application as part of usual farm practice.

## Methods

This study was approved by the Human Subjects Research Committee of the University of Minnesota and followed all applicable requirements and regulations. The target population was families living and working on an agriculture production operation where pesticides are used routinely. The methods for this study are described in detail elsewhere (Baker et al. 2005), and more information about the study is available on the study website (FFES 2004). Briefly, families were identified from lists of licensed pesticide applicators in Minnesota (*n* = 25,301) and South Carolina (*n* = 10,805). In addition to logistical considerations, Minnesota and South Carolina were selected as two states representing a diversity of agricultural practices, thus making study results more generalizable to other research. The licensed applicators were randomly ordered and contacted sequentially first by mail and then by telephone for recruitment into the study. Eligibility for the study required that *a*) the family lived on a farm, *b*) the family consisted of the farmer, a spouse, and at least one child 4–17 years of age, *c*) the farmer was planning to personally apply 2,4-D as part of the normal operation to at least 10 acres of cropland, some of which had to be within 1 mile, and on a contiguous piece of land with the family home, and *d*) the family members were willing to collect all of their urine for 5 days.

Eligible families were provided with comprehensive information about the study and taken through an informed consent process. The family received written information about the study procedures, risks, and benefits. This was followed by a meeting with a study representative who reviewed the protocol and answered any questions from the family. Signed consent was obtained from the applicator and spouse, a signed parental consent form was obtained for the children to participate, and signed assent forms were obtained from children 8–17 years of age and verbal assent was obtained for children < 8 years of age. A study start date was estimated based on predicted 2,4-D application date. The actual date of application was flexible to allow for changes brought on by weather and other needs of the farm. Participating families received $250 and an additional $50 for each child that participated. In addition, the cost of the chemical used in the study application was reimbursed up to a maximum of $1,000 (median reimbursement $207). All applications were conducted in the 2000 and 2001 growing seasons. Ultimately 34 applications of 2,4-D were observed, 17 in South Carolina and 17 in Minnesota.

The families were instructed to follow their normal routines related to pesticide application. The intent of the study was to measure exposure in real-world conditions, so no restrictions were made pertaining to the purchase of the chemical, when and how the farmers applied the chemical and maintained their equipment, how and when they changed or washed their clothes used in the application, whether the spouse and children were present when the application was made, and whether other chemicals were applied.

The farmer and spouse were asked to complete an enrollment questionnaire before and a follow-up questionnaire after the study application. The questionnaires emphasized the following: demographics, farm production and practices, pesticide application procedures, use of personal protective equipment, self-reported exposures including recent pesticide applications, activities at the time of the study pesticide application, and potential exposure to the children. The questionnaires were reviewed for completeness and participants were recontacted for missing information.

A research team member observed the chemical application and recorded the location and size of the field, proximity to the house, equipment used, chemical name and formulation, methods of mixing, personal protective equipment used, clothing worn, occurrence of spills, accidents, or repairs of the equipment, and presence of children, spouse, or pets during the mixing and application process.

The farmer, spouse, and participating children were asked to collect all urine voids for 5 days: the day before pesticide application through 3 days after pesticide application—hereafter referred to as days –1, 0, 1, 2, and 3. Each void was collected into 500-mL high-density polyethylene single-void containers. The family was provided with coolers and ice packs or a small refrigerator to store the urine. The date and time of each void was recorded by the study participants and logged by study staff when the urine specimens were picked up daily. If one or more of the family appeared to have low volumes or few voids, the farmer or spouse was asked to encourage the family to fully comply with the study protocol. The individual urine voids were refrigerated, combined proportionally by volume into 24-hr composite samples representing all urine voided in that period, and then frozen. The 24-hr urine composite samples were timed in 24-hr intervals based on the exact time the 2,4-D mixing and application process began on the day of application (day 0), including the 24 hr preceding initial contact (day –1) and the 3 24-hr periods following day 0 (days 1, 2, and 3).

2,4-D is excreted in the urine as 2,4-D or a 2,4-D conjugate (Sauerhoff et al. 1977). To estimate exposure, we analyzed the composite urine samples for 2,4-D, using a sensitive, selective method developed to measure both 3,5,6-trichloropyridinol (TCP; a metabolite of chlorpyrifos) and 2,4-D (Brzak and Bartels 2001). The analyte was hydrolyzed with concentrated hydrochloric acid to the nonconjugated form and extracted into toluene. The organic extracts were treated with *N*-methyl-*N*-(*tert*-butyl-dimethylsilyl)-trifluoroacetamide to form the *tert*-butyl-dimethylsilyl derivatives of 2,4-D. Analysis was accomplished by gas chromatography/mass spectrometry operating in the negative ion chemical ionization mode, and quantitation was performed using derivatized solvent standards. An isotopically labeled internal standard, ^13^C_6_-2,4-D, was used in the method. The intraday analysis of relative recovery, across the concentration range of 1–500 μ g/L, afforded relative recoveries of 85–90%. These results indicate minimal matrix effects for this assay. The limit of quantitation was 1 μ g/L. The results were corrected for laboratory fortification relative recovery results. No correction was made for recovery from field and travel spikes because the recoveries were just over 100% on average and the correction would only marginally decrease the estimated exposure.

Creatinine concentration in the 24-hr sample was measured and used to estimate the total creatinine in the urine collected over the 24-hr period. The creatinine analysis was performed by a spectrophotometric method and using a Kinetic Creatinine Procedure Kit provided by Data Medical Associates (Arlington, TX). The creatinine corrected concentration of 2,4-D was expressed as micrograms 2,4-D per gram total creatinine.

In addition to exposure assessment, we estimated systemic dose to aid risk characterization models. In risk characterization models, the relevant 2,4-D dose includes 2,4-D from all sources. The dose estimates for this study were based on total 2,4-D excretion over the entire postapplication period, which includes background concentration, exposure due to this application, and other sources. The daily total urine volumes were multiplied by the 2,4-D concentration from the composite sample to estimate daily exposure on the application day through day 3. We calculated a mean elimination rate for urinary 2,4-D from the applicator’s urinary 2,4-D data, using the sigma-minus method (Gibaldi and Perrier 1975), and this rate assumes that 93% of the absorbed 2,4-D is recovered in the urine (Sauerhoff et al. 1977).

We generated measures of central tendency (mean, median, range, and geometric mean) of the daily and the maximum urine 2,4-D concentrations for the applicators, spouses, and children to evaluate the data. A value of 0.5 μ g/L, the midpoint from 0 to the limit of detection (LOD; 1 μ g/L) was imputed for concentrations below the LOD. The distribution of concentrations was highly skewed; thus we used medians, geometric means, and geometric standard deviations to further characterize the central tendency of the exposures. We calculated the median change from baseline for daily 2,4-D urine concentrations and creatinine-corrected concentrations to characterize the exposure over the study period. We used 95% confidence intervals (CIs) to describe the precision of these estimates. We compared the differences in the baseline concentrations and estimated systemic dose between applicators, spouses, and children using both a nonparametric sign test to compare the differences in medians, and paired *t*-tests to compare the differences in the log of the doses; both analyses showed similar results. All analyses were conducted with PC SAS version 9.1 (SAS Institute Inc., Cary, NC).

Incomplete urine collections will introduce error in systemic dose estimates by underestimating the total amount of chemical excreted. To evaluate this error, we conducted a sensitivity analysis by imputing a floor volume for daily 24-hr urine samples. Using all 24-hr urine collections from the FFES (*n* = 1,895) (Baker et al. 2005), we assigned the 25th percentile of the volume for the applicators, spouses, and children 4–6, 7–9, 10–12, and ≥ 13 years of age to any 24-hr collection below that value. The systemic doses were recalculated using these adjusted volumes and compared to the original estimates.

Determinants of exposure are described by the geometric mean and standard deviation of the 24-hr sample with the highest postapplication 2,4-D concentration. Indicators of exposure for the children and spouses recorded by the observer included the closest distance from the field to the house, the number of acres treated, the number of loads, presence of the child or spouse during the application, and observation of opportunity for direct contact with the chemical (working with applicator, contact with equipment, or treated field). We evaluated self-report of washing the clothes used in the application as a determinant of exposure for the spouse. To evaluate potential differences by age, the children were stratified at 4–11 and ≥ 12 years of age, a cut point used in the Centers for Disease Control and Prevention’s (CDC) Third National Report on Human Exposure to Environmental Chemicals (CDC 2005). We evaluated the exposure to the applicator with reference to application practices. Categories were compared for the number of acres treated, number of loads (each time the tank was filled), observed skin contact, spills or accidents, any use of protective rubber gloves during mixing or application, repair of equipment, and tobacco use and eating during the application. These exposures were further evaluated by whether the applicator used gloves at any time during the application process. Single and multivariable generalized linear models were fit to examine statistical associations for linear and categorical exposure determinants and the natural log of the maximum daily concentration (SAS Institute Inc.).

## Results

The 34 licensed applicators applying 2,4-D were male (Table 1). Fifty-three children 4–17 years of age participated in the study. Two of the children were reported to have applied 2,4-D the week before the study application; however for one, a 5-year-old with no detectable 2,4-D preapplication, it was unclear whether this was correct because it was reported by only one parent and no date of use was given. No applicators or spouses reported mixing or applying 2,4-D before the study application date, but five had baseline concentrations substantially above the median (55, 63, 194, 199, and 230 μ g/L). All of these had substantial changes from baseline with maximum postapplication concentrations of 310, 686, 1,708, 2,236, and 439 μ g/L. Twenty children and eight spouses were present at some time during the application process, but only one spouse and four children were observed to have the opportunity for direct contact with the chemical during the application. Application methods were uniform for this chemical and were made with a boom sprayer; two applications also used hand wands at some time for portions of the application. The formulations were all liquid, either emulsifiable concentrate or aqueous solution and included both 2,4-D amine (*n* = 21) and ester (*n* = 13) formulations. The application acreages ranged from 10 to 281 and required 1–14 loads.

At least one urine void from all participants was available for the day before application and application day. One applicator and one spouse were missing samples for day 3. Seven samples were missing from children: day 1 (1), day 2 (1), and day 3 (5).

The median preapplication 2,4-D concentration was higher for applicators (2.1 μ g/L) than for children (1.5 μ g/L) and spouses (0.5 μ g/L—the LOD) (Table 2). The differences in the medians, though modest, were statistically significant by the sign test between the applicators and spouses (< 0.0001) and children (*p* = 0.0004), and spouses and children (*p* = 0.0013). The postapplication concentrations for the applicators were highly skewed and were much higher than those for children and spouses (Table 2). The geometric mean maximum concentration for the applicators was 64.2 μ g/L and occurred on the day after application. There was not an apparent peak for children and spouses, and the geometric means varied little across the study period. However, the highest concentration for a child (640.4 μ g/L) occurred on day 1 in a child who assisted with the application. The median change from baseline was highest (74.7; 95% CI, 25.1–103.6) for the applicators on day 2. The concentrations in the spouse following application were not statistically different from baseline. The change from baseline for the children was highest on day 3 (1.5 μ g/L; 95% CI, 0.8–3.5). The relative patterns were similar for creatinine corrected urine 2,4-D concentrations. The results expressed as micrograms analyte per gram creatinine are presented to allow comparison to other research addressing similar age and sex categories.

2,4-D was detectable in 70% of the applicators, 62% of the children, and 41% of the spouses at baseline (Table 3). The results were similar between older and younger children. The postapplication maximum concentration for the children 4–11 years of age was somewhat higher than for children ≥ 12 years of age. The geometric mean estimated systemic dose over the period of the study was 2.46, 0.08, and 0.22 μ g/kg body weight for the applicators, spouses, and children, respectively (Table 3). The geometric mean dose for the younger children was nearly 3-fold greater than for the older children; however, the range of the doses for the older children was much greater, with a maximum dose of 31.07 versus 7.16 μ g/kg, which is attributable to contact with the application process. Eight applicators and one child exceeded 10 μ g/kg. The sensitivity analysis that set a minimum volume for each 24-hr period did not change the overall distribution. The adjusted geometric means were 2.52, 0.09, 0.32, and 0.12 μ g/kg body weight for the applicators, spouses, children 4–11 years of age, and children ≥ 12 years of age, respectively.

The log of the maximum postapplication urine 2,4-D concentrations was correlated with the number of acres treated for the spouses (β = 0.0099, *p* = 0.03) and older children (β = 0.0199, *p* = 0.004), but not in the younger children (β = 0.0052, *p* = 0.17) (Table 4). This association remained among spouses and children who were not present at the application. Spouses who were present at some time during the application had higher urine 2,4-D concentrations than those not present (β = 0.779, *p* = 0.094), and the one spouse who was observed to potentially have direct contact with the process had a higher urine 2,4-D concentration (18.2 μ g/L). Children who were present at any time during the application had higher exposure than those who were not (geometric mean = 9.6 vs. 3.3 μ g/L; *p* = 0.011). The concentrations varied by sex as well as age in the children, with the younger girls and older boys having higher concentrations (Table 4), but the concentrations were not statistically different.

The number of acres, number of loads, observed skin contact, and repairing equipment during the application were all positively associated with urine 2,4-D concentrations among the applicators (Table 5). The use of gloves during the mixing and application process reduced exposure dramatically, with the geometric mean urine concentration for applicators not wearing gloves > 7-fold greater (236 vs. 44 μ g/L). The use of tobacco, eating during the application, not having a closed cab, and washing the equipment were not associated with exposure in the univariate analysis. The difference in exposure by glove use was observable across all covariates; applicators who wore gloves had consistently lower urine 2,4-D concentrations (Table 6). Multivariate models determined that three covariates—wearing gloves, acres treated, and repairing equipment—were consistently predictive of exposure (Table 7). Some covariates, such as glove use and observed skin contact, were highly correlated and could not be included in the model together. The number of loads, though correlated with the number of acres, was not predictive in the multivariate models, despite being an indicator of increased potential for direct contact with the chemical.

## Discussion

In this study, the application of 2,4-D to crop land resulted in exposure to the applicator and some other family members, but the magnitude of exposure is determined by the potential for direct contact with the application process or chemical. Overall, exposure to the children and spouses was low, and was minimal or below detection for those who did not have contact with the application process. Some putative determinants of exposure to applicators were predictive of exposure—primarily use of gloves, size of application, and having to repair equipment—but the effect of other commonly cited exposure modifiers could not be distinguished. These results have implications for pesticide exposure assessment and risk characterizations for farm families. The results also emphasize recognized exposure pathways that can be modified to reduce exposure: specifically reducing children’s potential for contact with the application process.

Most of the applicators and children had detectable urine concentrations of 2,4-D at baseline, whereas the spouses had a somewhat lower frequency of detectable concentrations. These baseline concentrations differ from other published population-based urine concentrations. The 2001–2002 National Health and Nutrition Examination Survey measured 2,4-D in spot samples of participants throughout the country and reported detectable concentrations in children 6–11 and 12–19 years of age; adult males had detectable concentrations at the 75th percentile (0.3, 0.25, and 0.1μ g/L respectively) and adult females at the 90th percentile (0.48 μ g/L) (CDC 2005). Curwin et al. (2005) reported similar prevalence of detectable 2,4-D urine concentrations in farmers who had not applied 2,4-D. The geometric mean concentrations for farmers who applied 2,4-D were lower than those in our study, but the timing of the samples in relation to application did not always capture the peaks in excretion (Curwin et al. 2005). Compared to a study of farm families in Ontario, Canada (Arbuckle et al. 2004), families in our study had a higher prevalence of detectable concentrations in children (60 vs. 9.8%) and a higher maximum concentration in children (640 vs. 100 μ g/L). The differences in background concentrations could be attributable to sampling methods, calendar period of exposure characterization, or higher environmental concentrations. The urine samples for the FFES were all collected in the spring and early summer when use of the chemical is at its highest for agricultural and nonagricultural purposes. The LOD for our study and that of Arbuckle et al. (2004) are equivalent (1 μ g/L), and modestly higher than the CDC method (0.2 μ g/L) (2005); thus the difference in prevalence of exposure is unlikely attributable to laboratory methods. Higher background levels in farm families are also plausible as a result of low-level contamination of the living area. A study of households in a corn and soybean region of Iowa reported 2,4-D to be the most prevalent of several herbicides in house dust samples, and the concentrations of all herbicides was associated with proximity to crops (Ward et al. 2006). The houses from a subsample of participants in the Agricultural Health Study in Iowa had 2,4-D detected in all house dust samples, and the concentration was associated with spray applications (Curwin et al. 2005). The Ontario Farm Family Health Study also reported residual contamination of several household surfaces with 2,4-D (Arbuckle et al. 2006).

The collection of sequential 24-hr urine samples allowed estimation of absorbed dose over the study period. Exposure and dose are correlated, and exposure estimates based on biological monitoring are useful for reconstruction of exposure for an epidemiologic study. Risk characterization, however, requires a more complete understanding of absorbed dose. The reference dose (RfD) for 2,4-D established by the U.S. Environmental Protection Agency (EPA) is 0.01 mg/kg/day, based on a no observed adverse effect level of 1.0 mg/kg/day from a 2-year bioassay of rats (U.S. EPA 1988). We estimated dose from all 2,4-D excreted in the urine over 4 days postapplication. With the assumption that all exposure from the day of application is represented in this estimate, eight applicators and one child, who assisted in the application, exceeded the daily reference dose for that day. The application of herbicides occurs only a few days out of the year, so it is difficult to place these rare excursions above the RfD within the prevailing risk characterization paradigm based on a daily dose over a lifetime. These dose estimates do, however, provide baseline information for risk characterization based on an actual use scenario.

The peak urine 2,4-D concentration in applicators occurred on the day after application. This peak in excretion is expected given an approximately 17 hours biological half-life of 2,4-D following oral administration (Sauerhoff et al. 1977), and verifies that the applicators are receiving most of their exposure during application. The lower maximum concentrations resulted in less dramatic peak exposures in the spouses and children. Although the children directly exposed to the chemical during the application had their greatest exposure at that point, other children and spouses may have acquired secondary exposure over a course of days; however, these exposures were too low to evaluate critically. Understanding the excretion profiles of herbicides is useful for establishing future biomonitoring protocols for exposure characterization. The FFES has demonstrated that two of the most commonly used herbicides—glyphosate (Acquavella et al. 2004) and 2,4-D—have very different excretion profiles in farm families. To properly ascertain maximum exposure in pesticide biomonitoring protocols, the chemical specific characteristics, including the pharmacokinetics and timing of peak excretion, need to be considered. This applies to protocols using either single void or 24-hr urine collections.

Several predictors of applicator exposure were correlated with urine 2,4-D levels, most of which are surrogates for potential for direct dermal contact. The number of acres treated and the number of loads are surrogates for the size of application and the opportunity for chemical contact, but also are highly correlated. Both were associated with exposure in the univariate analysis, but only acres remained a predictor of exposure in the multivariate analysis. The use of gloves during mixing, loading, or application reduced exposure and modified the exposure levels in other covariates. Identifying consistent predictors of exposure for herbicide applications is difficult. Arbuckle et al. (2002) also reported glove use, hours using 2,4-D, and tank capacity to be associated with urine 2,4-D levels in applicators; these are comparable to the predictive skin contact and application size surrogates that we report. Interestingly, their predictive model for 4-chloro-2-methylphenoxyacetic acid applications was different, although the application methods were similar. Use of gloves was also predictive of exposure following boom spray applications of glyphosate (Acquavella et al. 2004) but not for chlorpyrifos (Alexander et al. 2006) in the FFES. The varying exposure scenarios for different pesticides emphasize the need to consider not only the use of the chemical, but also the type of chemical and formulation in exposure estimations.

The FFES is a comprehensive exposure study, but is not without limitations. Overall, the compliance with the urine collection protocol was good, but a few of the 24-hr urine collections were incomplete (Baker et al. 2005). Although the 2,4-D concentrations may be well represented in these incomplete samples, the systemic dose will be underestimated. The applications in this study were intended to represent the real-world scenarios that a farm family would encounter year in and year out; however, the study was limited to a single application. The applicators were asked to follow their usual practices to capture real-world exposure scenarios, but it is possible that some altered practices because the field observer was present. Changes in equipment or agricultural practices in the past or future may alter the actual exposure to the family. For epidemiologic studies, this will be a problem largely for reconstructing exposure in the distant past. We recognize that intraindividual variation in exposure may be as large as or larger than interindividual exposure (Kromhout and Vermeulen 2001). This study was not able to address how much the exposure will vary over one or more seasons and whether the peak exposures are representative of exposures for that individual or the distribution of expected exposure from applying this chemical.

The strengths of the FFES include the collection of serial 24-hr urine samples, allowing estimation of systemic dose, and adherence of the farmers to usual practice in this exposure characterization. This builds on the work of Arbuckle and colleagues in establishing parameters for herbicide exposure (Arbuckle et al. 2002, 2004), and will aid the development and interpretation of exposure models for epidemiologic studies of pesticide-exposed populations (Acquavella et al. 2006; Dosemeci et al. 2002).

## Figures and Tables

**Table 1:** Characteristics of farm families that applied 2,4-D in the FFES (no.).

Characteristic	Applicators	Spouse	Children
Sex			
Male	34	0	31
Female	0	34	22
Age (years)		
Mean (range)	43.6 (31–58)	40.2 (30–60)	10.1 (4–17)
Mix or apply 2,4-D in previous week^a^		
Yes	0	0	2
No	34	34	51
Present during application^b^		
Yes	34	8	20
No	0	26	33
Opportunity for direct contact with chemical^b^^,^^c^		
Yes	16	1	4
No	18	33	49

a As reported on applicator and spouse enrollment questionnaire.

b Reported by field observer.

c Skin contact for applicator; for children and spouse working with applicator, contact with equipment, or treated field.

**Table 2:** Summary of urine 2,4-D concentrations by volume and per gram creatinine by study day with change from baseline.

	Applicators		Spouses		Children	
Study day	μ g/L^a^	μ g/g^b^	μ g/L	μ g/g	μ g/L	μ g/g
Preapplication						
No.	34	34	34	34	53	53
Median^c^	2.1	1.5	0.5	0.5	1.5	1.1
Range (minimum–maximum)	0.5–230.9	0.1–130	0.5–20.4	0.5–20.4	0.5–53.2	0.1–48.6
GM	3.8	2.1	1.0	0.9	1.4	1.2
Application						
No.	34	34	34	34	53	53
Median	21.3	17.1	0.5	0.8	1.8	1.6
Range (minimum–maximum)	0.5–452.6	0.4–148	0.5–15.9	0.2–21.6	0.5–336.2	0.2–190.9
GM	29.1	16.3	1.0	0.9	2.1	1.9
Median change from baseline (95% CI)	17.8 (11.0–62.8)	13.9 (6.6–28.4)	0.0 (0.0–0.5)	0.0 (−0.2–0.3)	0.0 (0.0–0.9)	0.3 (0.0–0.7)
Day 1						
No.	34	34	34	34	52	52
Median	73.1	45.8	1.2	1.1	2.9	2.3
Range (minimum–maximum)	1.5–1856.0	1.1–533.8	0.5–20.0	0.2–13.1	0.5–640.4	0.3–660.2
GM	64.2	33.4	1.3	1.2	3.6	3.1
Median change from baseline (95% CI)	70.7 (31.7–123.4)	43.0 (13.4–77.7)	0.0 (0.0–1.2)	0.2 (−0.2–0.8)	2.1 (0.0–3.0)	0.8 (0.3–2.8)
Day 2						
No.	34	34	34	34	52	52
Median	80.2	37.5	1.3	0.9	3.4	2.6
Range (minimum–maximum)	0.5–2236.0	0.4–822	0.5–24.9	0.2–16.3	0.5–263.3	0.3–135.4
GM	45.3	23.7	1.4	1.2	3.5	2.9
Median change from baseline (95% CI)	74.7 (25.1–103.6)	33.3 (10.3–70.5)	0.1 (0.0–0.7)	0.1 (−0.1–0.7)	1.6 (0.6–3.5)	0.8 (0.6–2.3)
Day 3						
No.	33	33	33	33	48	48
Median	34.3	21.3	0.8	0.9	3.0	3.0
Range (minimum–maximum)	0.5–1529.2	0.4–580	0.5–15.9	0.5–8.8	0.5–97.9	0.3–117.8
GM	28.3	16.2	1.3	1.1	3.4	3.0
Median change from baseline (95% CI)	27.3 (10.8–54.1)	16.3 (6.8–36.1)	0.0 (0.0–0.7)	0.0 (−0.2–0.5)	1.5 (0.8–3.5)	1.6 (0.7–3.2)

GM, geometric mean.

a μ g/L 2,4-D concentration in urine.

b μ g/g 2,4-D per gram creatinine.

c Sign test for difference in medians between the applicators and spouses (< 0.0001), applicators and children (*p* = 0.0004), and spouses and children (*p* = 0.0013).

**Table 3:** Maximum daily 2,4-D concentrations^a^ and estimated 2,4-D dose^b^ across all study days for all participants by farm family member.

			Children		
	Applicator	Spouse	All	4–11 years	≥ 12 years
No.	34	34	53	33	20
Detectable preapplication (%)	70.6	41.2	62.3	63.6	60.0
Detectable any day (%)	100.0	67.6	88.7	90.9	85.0
Maximum urine 2,4-D (median)	90.9	1.7	4.7	6.5	1.9
Concentration (μ g/L)					
Range	1.5–2236.0	0.5–24.9	ND–640.4	0.5–109.9	0.5–640.4
GM	71.9	1.7	4.9	6.4	3.2
GSD	6.2	3.2	4.5	4.0	5.1
Dose (μ g/kg)^b^					
GM	2.46	0.08	0.22	0.32	0.12
All days					
GSD	5.66	2.59	4.60	3.50	5.92
Maximum	58.48	1.14	31.07	7.16	31.07
90th percentile	23.99	0.25	1.07	1.07	1.44
75th percentile	9.28	0.16	0.46	0.53	0.20

Abbreviations: GM, geometric mean; GSD, geometric standard deviation; ND, not detectable.

a μ g/L 2,4-D LOD was assigned to 0.5 μ g/L.

b U.S. EPA RfD = 0.01 mg/kg/day, or 10 μ g/kg/day. *p*-Value for difference in log of dose between farmer and all children, farmer and children 4–11 years of age, farmer and spouse, spouse and children, and spouse and children 4–11 years of age: *p* < 0.0001; for spouse and children ≥ 12 years of age, *p* = 0.32.

**Table 4:** Geometric means and standard deviations of the maximum urine 2,4-D concentration from 24-hr samples for spouses and children 4–11 and ≥ 12 years of age by potential exposure surrogate.

				Children (age)					
	Spouses			4–11			≥ 12		
Exposure metric	No.	GM	GSD	No.	GM	GSD	No.	GM	GSD
State									
Minnesota	17	2.0	3.1	19	7.3	2.9	11	3.6	3.2
South Carolina	17	1.5	3.3	14	5.3	5.7	9	2.8	8.5
Sex									
Female	34	1.7	3.2	18	8.5	3.0	13	2.7	6.6
Male				15	4.5	5.1	7	4.5	2.9
Distance from house (yards)									
< 75	9	1.6	3.5	5	5.0	2.7	8	3.6	9.1
75–175	8	3.1	2.8	7	6.1	5.1	5	3.99	3.8
175–600	8	1.8	2.8	11	8.2	2.1	4	4.1	3.4
> 600	9	1.0	3.3	10	5.6	7.1	3	1.3	2.7
Acres treated									
< 25	9	1.2	3.7	8	5.5	9.5	7	1.8	2.9
25–< 50	3	1.1	2.1	4	2.2	3.0	1	19.8	
50–< 75	7	1.9	2.4	5	12.8	3.9	5	2.3	1.8
≥ 75	15	2.3*	3.4	16	7.2	2.0	7	5.7*	10.7
Loads mixed and applied									
1–2	9	1.1	2.6	8	4.1	4.8	5	4.1	3.1
3–5	14	1.8	3.7	16	7.4	4.4	8	2.1	3.4
≥ 6	11	2.3	2.9	9	7.3	2.8	7	4.4	10.3
Present at some time during application									
Yes	8	3.1	2.9	12	11.5	4.1	8	7.3	8.8
No	26	1.4	3.1	21	4.6	3.6	12	1.9	2.4
Direct contact opportunity^a^									
Yes	1	18.3		2	108.6*	1.02	2	17.9*	157.5
No	33	1.6	3.0	31	5.3	3.4	18	2.7	2.8
Wash pesticide application clothes									
Yes	22	1.8	3.1						
No	12	1.6	3.4						

Abbreviations: GM, geometric mean; GSD, geometric standard deviation.

a Observed by field observer; working with applicator, contact with equipment, or treated field.

* *p* < 0.05 highest to lowest category.

**Table 5:** Selected determinants of the maximum urine 2,4-D concentration from 24-hr samples for applicators.

Exposure metric	No.	GM	GSD	*p*-Value
State				
Minnesota	17	76.4	7.9	0.851
South Carolina	17	67.7	5.0	
Acres treated				
< 25	9	57.0	5.2	0.007^a^
25–< 50	3	12.9	4.9	
50–< 75	7	100.9	7.5	
≥ 75	15	99.7	6.3	
Loads mixed and applied				
1–2	9	53.0	7.1	0.023^a^
3–5	14	50.5	7.4	
≥ 6	11	144.8	4.0	
Skin contact observed				
Yes	16	188.8	4.3	0.002
No	18	30.5	5.7	
Closed cab on tractor				
Yes	15	61.4	9.1	0.66
No	19	81.5	4.6	
Any spill or accident observed				
Yes	12	115.4	6.7	0.27
No	22	55.6	5.8	
Wore rubber gloves during process				
Yes	24	43.8	5.9	0.01
No	10	236.2	4.0	
Repaired equipment during application				
Yes	13	184.1	3.6	0.016
No	21	40.2	6.7	
Reported washing application equipment				
Yes	21	69.4	6.4	
No	13	76.2	6.5	0.880
Used tobacco during application				
Yes	11	106.7	5.3	0.392
No	23	59.6	6.7	
Ate during application				
Yes	4	140.1	3.8	0.44
No	30	65.8	6.6	

Abbreviations: GM, geometric mean; GSD, geometric standard deviation.

a Linear trend: natural log of the 2,4-D concentration and the number of acres or loads.

**Table 6:** Selected determinants of the maximum urine 2,4-D concentration (μ g/L) from 24-hr samples for applicators by use of protective rubber gloves.

	No			Yes		
Exposure metric	No.	GM	GSD	No.	GM	GSD
Acres treated						
< 25	3	347.0	1.9	6	23.1	3.1
25–< 50	0	—	—	3	12.9	4.9
50–< 75	1	202.4	—	6	89.9	8.9
≥ 75	6	199.9	6.0	9	62.7	6.2
Loads mixed and applied						
1–2	1	207.8	44.7	8	44.7	7.6
3–5	2	621.7	33.2	12	33.2	6.2
≥ 6	7	182.4	96.7	4	96.7	3.4
Skin contact observed						
Yes	7	329.9	3.8	9	122.4	4.3
No	3	108.2	4.2	15	23.7	5.6
Any spill or accident						
Yes	5	409.9	3.1	7	46.7	6.9
No	5	136.1	4.6	17	42.7	5.9
Repaired equipment during application						
Yes	6	247.6	3.3	7	142.8	4.1
No	4	220.0	6.4	17	26.9	5.6
Used tobacco during application						
Yes	6	211.7	4.3	5	46.9	5.4
No	4	278.2	4.5	19	43.0	6.4
Ate during application						
Yes	1	292.9	—	3	109.6	4.6
No	9	230.6	4.4	21	38.5	6.1

Abbreviations: —, GSD not calculated; GM, geometric mean; GSD, geometric standard deviation.

**Table 7:** Final regression models for predictors of the log of the maximum urine 2,4-D concentration for the applicator.

Covariate, metric	β	SE	95% CI	*p*-Value
Wore rubber gloves				
Yes	Referent			
No	1.0108	0.58548	−0.18489–2.20654	0.0945
Repaired equipment				
No	Referent			
Yes	1.2238	0.53906	0.12295–2.32475	0.0305
Acres treated				
Acre	0.01263	0.00453	0.00336–0.02189	0.0092

*R*^2^ = 0.4208.
